# Retrospective analysis of deformed complex vertebral osteotomy in children with severe thoracic post-tubercular angular kyphosis

**DOI:** 10.1186/s12891-022-05756-1

**Published:** 2022-08-23

**Authors:** Hong-Qi Zhang, Ang Deng, Chao-Feng Guo, Qi-Le Gao, Emmanuel Alonge

**Affiliations:** 1 Department of Spine Surgery and Orthopaedics, Xiangya Hospital, Central South University, Xiangya Road 87, Changsha, 410008 China; 2 National Clinical Research Center for Geriatric Disorders, Xiangya Hospital, Central South University, Xiangya Road 87, Changsha, 410008 China

**Keywords:** Wedge osteotomy, Deformed complex vertebrae, Post-tubercular, Angular kyphosis, Children

## Abstract

**Background:**

Many surgical options have been described to manage post-tubercular kyphosis, but the standard approach for treating severe post-tubercular angular kyphosis in children has not been established yet. The present study was performed to evaluate the safety and efficacy of deformed complex vertebral osteotomy (DCVO) for the treatment of severe thoracic post-tubercular angular kyphosis (> 70°) in children.

**Methods:**

Deformed complex vertebrae indicated that multiple deformed and fused vertebrae were usually involved with two or more vertebral bodies and the partial or total fusion of many segments' facet joints and intervertebral discs. Thus, DCVO indicated that a wider posterior wedge-shaped and three-column osteotomy was performed within deformed complex vertebrae to correct a more extensive range of angles. From 2010 to 2017, 15 children who suffered from severe thoracic post-tubercular angular kyphosis underwent DCVO. Deformed complex vertebrae involved two vertebral bodies in 9 patients and three vertebral bodies in 6 patients. The Visual Analogue Scale (VAS) and Oswestry Disability Index (ODI) were assessed preoperatively and at the final follow up. This was a retrospective study analysing the outcome after grade 4/5 spinal osteotomies in deformed complex vertebrae.

**Results:**

The mean duration of surgery was 239 ± 37.81 min. The average period of follow-up was 31.6 ± 6.98 months. The preoperative mean kyphosis of deformed complex vertebrae was 83.39° ± 9.04°; the mean thoracic kyphosis (TK) and lumbar lordosis (LL) were 81.09° ± 8.51° and 80.51° ± 7.64°, respectively; the mean sagittal vertical axis (SVA) was 3.83 cm ± 1.43 cm. The postoperative mean kyphosis of deformed complex vertebrae was reduced to 19.98° ± 2.47° (*P* < 0.001) with a mean kyphosis correction of 63.41°; at the final follow up, it was 18.4° ± 2.29° (*P* < 0.001) without obvious loss of correction. The postoperative mean TK, LL, and SVA were reduced to 24.05° ± 3.84°, 46.9° ± 3.53°, and 0.6 cm ± 0.34 cm, respectively (*P* < 0.001 for all); and there was no obvious loss of sagittal alignment and balance at the final follow up (*p* = 0.982, *p* = 0.604, *p* = 0.754). Complicated with neural dysfunction preoperatively, 5 Frankel's grade D cases showed complete neurological recovery at final follow up. VAS score reduced from 3.6 ± 1.18 to 0.87 ± 0.64 (*P* < 0.001); and ODI score reduced from 22.21 ± 6.93 to 5.02 ± 2.6 (*P* < 0.001) at the final follow up.

**Conclusions:**

DCVO was an individualized osteotomy for treating severe thoracic post-tubercular angular kyphosis in children and could be safe and effective in reducing the incidence of complications and significantly improving kyphosis correction.

## Background

Spinal tuberculosis accounts for half the cases of osteo-articular tuberculosis. Vertebral inoculation occurs via the haematogenous route, and the process then spreads to the intervertebral disk and, in some cases, to the adjacent vertebra. Para-spinal abscesses may develop by direct spread from the vertebral lesion. The thoracic spine is predominantly involved [1]. The patients with spinal tuberculosis that were treated conservatively presented with kyphosis with an average of 15°, and eventually, 3–5% of them ended up with a kyphotic angle of more than 60° [2–5]. Furthermore, even if spinal tuberculosis heals during the period of growth and development of children, an imbalance in the spinal growth of the anterior and posterior columns could still aggravate kyphosis [6, 7]. Severe post-tubercular angular kyphosis generally affects children's self-confidence and appearance and could even lead to severe neurological impairment and cardiopulmonary dysfunction. Many surgical options have been described to manage post-tubercular angular kyphosis, but the standard approach has not yet been established.

Until now, surgical osteotomy has been the leading choice for correcting the deformity and realigning the spine. Posterior column osteotomy (PCO) is not indicated as a first-line treatment for post-tubercular angular kyphosis because of the difficulty in decompressing the spine canal anteriorly and the relatively small correction angle [8–10]. Conversely, pedicle subtraction osteotomy (PSO) is a wedge-shaped resection in one vertebra which includes the partial vertebral body and posterior column elements and can only acquire correction of 30°-40° [9–11]. Vertebral column resection (VCR) requires removing one or several whole vertebrae, concurrently including adjacent discs and a portion of the ribs in the thoracic region, which could significantly correct severe kyphoscoliosis. However, VCR has several disadvantages, such as neurological complications, blood loss, operation time, nonunion or pseudoarthrosis, and a significant increase in the incidence of postoperative complications [12–15]. Therefore, the present study was performed to evaluate the safety and efficacy of modified posterior wedge osteotomy within deformed complex vertebrae to treat severe thoracic post-tubercular angular kyphosis (> 70°) in children. This was a retrospective study analysing the outcome after grade 4/5 spinal osteotomies.

## Materials and methods

### Patient data

In this study, we retrospectively evaluate the clinical efficacy of 15 children (8 boys and 7 girls; age, 5–11 years; average age, 7.8 ± 2.0 years) that suffered from severe thoracic post-tubercular angular kyphosis and were treated in our department between 2010 to 2017. In all children, X-ray, CT, and MRI examinations revealed: deformed complex vertebrae involved with two vertebral bodies in 9 patients and three vertebral bodies in 6 patients; the lesions healed completely in all 15 cases without further destruction of the vertebral bodies or abscesses.

All cases presented with apparent kyphotic deformity and persistent back pain, and some of them incurred incomplete paralysis. C-reactive protein (CRP) and erythrocyte sedimentation rate (ESR) in all cases were normal. The Frankel scoring system was used to assess neurological function. Frankel's grade D was in 5 patients, and Grade E in 10 patients. The Ethics Committee of Xiangya Hospital of Central South University approved the study. All methods were performed by following the relevant guidelines and regulations. Written informed consent was acquired from each patient (or their parents and legal guardians) to authorize treatment, imaging studies, and photographic documentation. The patients (or their parents or legal guardians) permitted their photographs and anonymised clustering data for publication.

The inclusion criteria were based on the following criteria: (1) severe thoracic angular kyphosis more than 70°; (2) the tuberculosis lesions completely healed; (3) CRP (range, 0—8 mg/L) and ESR (range, 0—21 mm/h) were normal; (4) deformed complex vertebrae involved two or more vertebral bodies; (5) children of elementary school age; (6) patients treated at our hospital with a minimum 2-year follow-up.

Exclusion criteria were based on: (1) no severe or progressive kyphosis; (2) deformed vertebra involved with only one vertebral body; (3) spine deformities caused by congenital malformations or trauma; (4) incomplete clinical and radiological documentation at follow-up.

### Operative procedure

During the operation, somatosensory evoked potential (SEP) and motor evoked potential (MEP) were thoroughly utilized to monitor the spinal cord functions. After exposure of posterior spinal components through a midline incision, pedicle screws were placed in one or two segments above and below deformed complex vertebrae. The ribs in affected segments were resected enough to reveal the lateral aspect of deformed complex vertebral bodies. The laminae and facet joints were removed to expose the spinal canal and nerve root canal. The nerve roots were identified and preserved. Isolation and decompression of the nerve roots were acquired to reduce the tension of the spinal cord and dura. If intraoperative exposure and osteotomy suffer from difficulties, corresponding nerve roots should be ligated appropriately. The temporary rod adhered to screws alternately. Deformed complex vertebral osteotomy (DCVO) was performed at the apex of kyphotic deformity. Pre-bent rods were used to gently and progressively replace the temporary rod. Compression was applied to the screws and rods to achieve bone-to-bone closure. Laminectomy was performed on the adjacent regions of the normal vertebrae when the dura was folded. The shorter spinal cord could be further decompressed by increasing the inner diameter of the spinal canal. For fusion, allogeneic or autogenous bone grafts could be used.

**Fig. 1:**
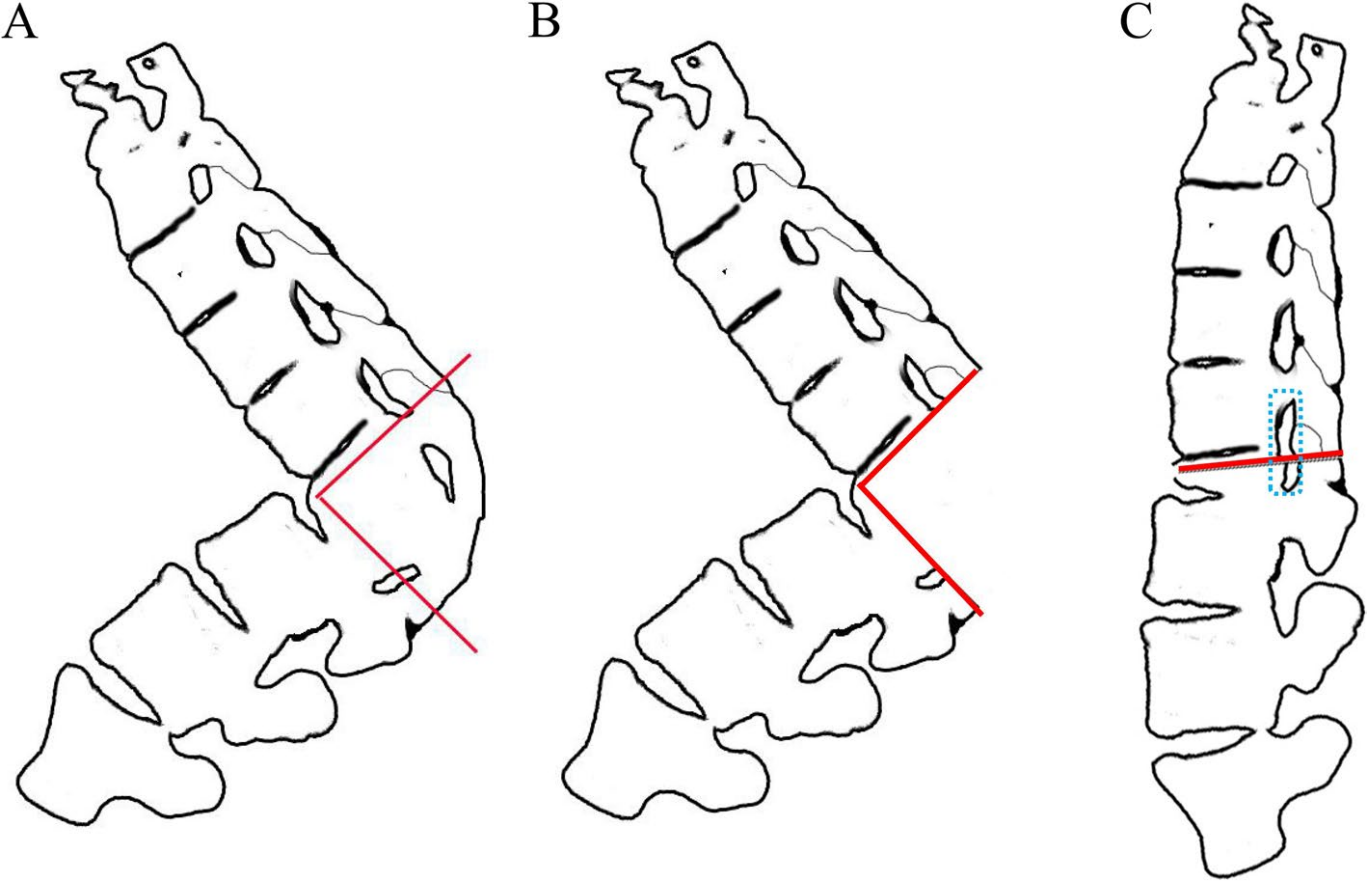
Diagram of the DCVO. **A** a wider posterior wedge-shaped and three-column osteotomy was performed within multiple deformed complex vertebrae. **B** The upper and lower endplates were preserved. Anterior longitudinal ligament was also preserved as a hinge for closure of the gap. **C** bone-to-bone closure was achieved in the anterior and middle columns without interbody fusion cages, resulting in better fusion efficiency. The part surrounded by a blue dotted line was prepared to avoid root entrapment through decompression and expanding the inner diameter of the intervertebral foramen

Deformed complex vertebrae indicated that multiple deformed and fused vertebrae were usually involved with two or more vertebral bodies and the partial or total fusion of many segments' facet joints and intervertebral discs [16]. The DCVO involves performing a larger posterior wedge-shaped and three-column osteotomy within deformed complex vertebrae using an ultrasonic scalpel, osteotomes, and high-speed drilling to correct a wider range of angles. The upper and lower endplates of deformed complex vertebrae that adjoined normal vertebrae were preserved. Special attention was taken not to break the anterior longitudinal ligament, which served as a hinge when closing the gap. Bone-to-bone closure and fusion were achieved without the interbody fusion cages, resulting in a better fusion efficiency (Fig. 1).

### Visual Analogue Scale (VAS) and Oswestry Disability Index (ODI)

According to self-assessment, the pain was measured preoperatively and at the final follow up by VAS without an analgesic. Furthermore, the daily activities were also assessed preoperatively and at the final follow up by ODI to analyse clinical function.

### Radiographical and statistical analysis

At the preoperative, postoperative, and final follow-up stages, radiographs' parameters, including kyphosis of deformed complex vertebrae, TK, LL, SVA, were measured. The data were shown as means ± SD and analysed using SPSS 22.0. Paired t-test was used to compare the parameters preoperatively, postoperatively, and at the final follow up. *P* < 0.05 indicates statistically significant difference.

## Results

### Surgical results

The mean duration of surgery was 239 ± 37.81 min (range, 170—310 min) and the mean blood loss was 641.33 ± 185.12 ml (range, 410—1010 ml). A thorough neurological examination was carried out in all of the cases after the operation and at the final follow up. During operation, no severe complications such as extensive vessel injury, spinal cord injury, or nerve injury occurred. Moreover, there were no cerebrospinal fluid leakage, death, or severe infection cases, and none showed new irreversible neural injury. The average period of follow-up was 31.6 ± 6.98 months (range, 24—48 months). No complications related to instrumentation failure occurred. Two cases experienced MEP changes that were reduced by more than 50% of baseline amplitude. However, the intraoperative wake-up test was successful in all of them. Complicated with neural dysfunction preoperatively, 5 Frankel's grade D cases showed complete neurological recovery at final follow up.

### Kyphosis correction

**Fig. 2:**
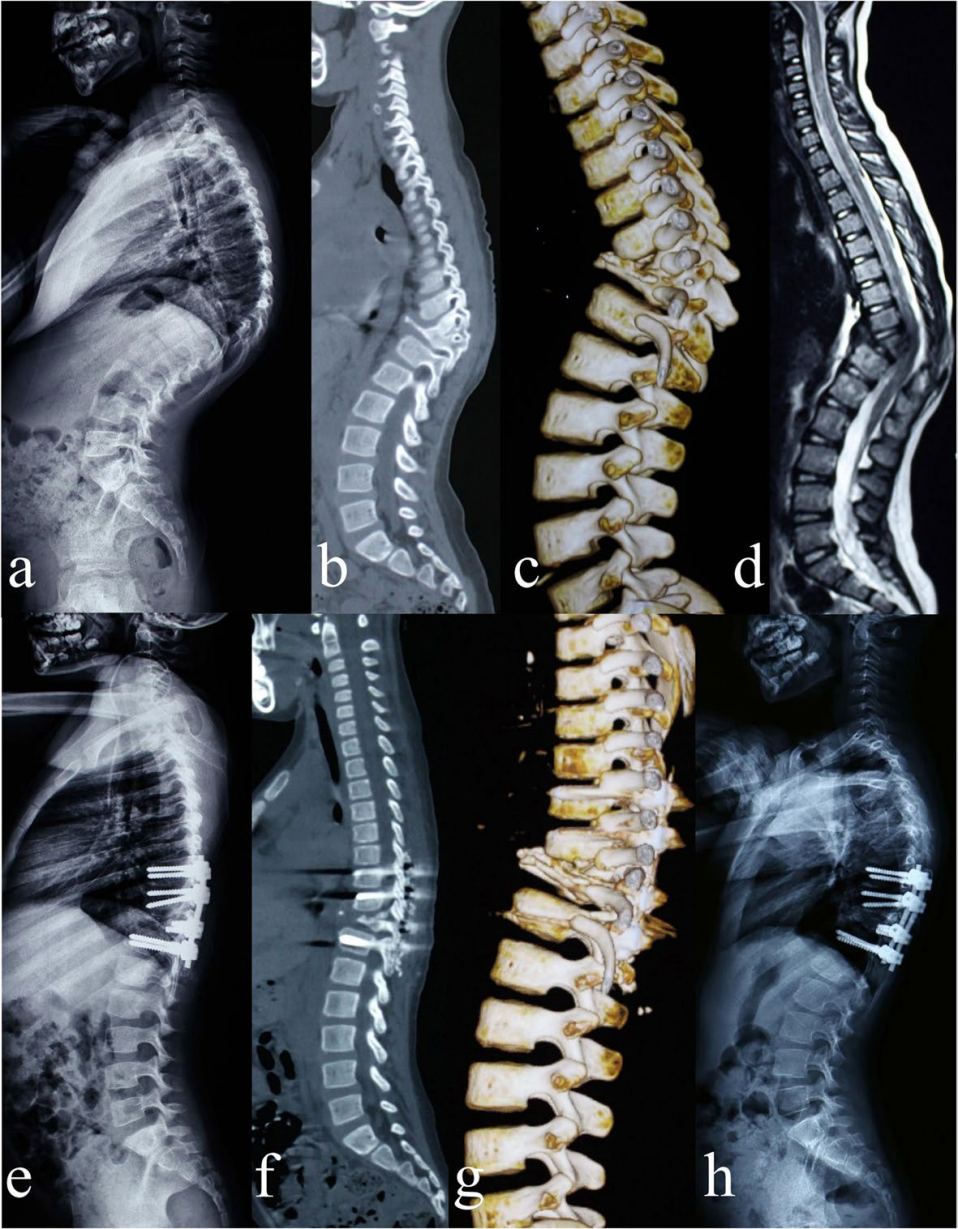
A 5-year-old girl with severe thoracic post-tubercular angular kyphosis. **a-d** Preoperative X-ray, CT, and MRI showed post-tubercular deformed complex vertebrae involved with T9-11. The angular kyphosis of deformed complex vertebrae was 81.2°, TK was 91.3°, LL was 85.7°, SVA was 4.3 cm, and the spinal cord was compressed obviously. **e–g** After DCVO, postoperative X-ray and CT showed that the kyphosis of deformed complex vertebrae was reduced to 20.3° with kyphosis correction of 60.9°, TK was 27.5°, LL was 47.9°, and SVA was 0.9 cm. **h** Postoperative radiographs at 30 months after surgery showed that the kyphosis of deformed complex vertebrae was further reduced to 18.4°, TK was 29.8°, LL was 55.3°, and SVA was 1.4 cm

The preoperative mean kyphosis of deformed complex vertebrae was 83.39° ± 9.04° (range, 70.8°-102.6°); the mean thoracic kyphosis (TK) and lumbar lordosis (LL) were 81.09° ± 8.51° (range, 66.1°-97.4°) and 80.51° ± 7.64° (range, 68.5°-93.8°), respectively; the mean sagittal vertical axis (SVA) was 3.83 cm ± 1.43 cm (range, 1.7 cm-6.8 cm). The postoperative mean kyphosis of deformed complex vertebrae was reduced to 19.98° ± 2.47° (range, 15.7°-23.6°) with a mean kyphosis correction of 63.41°, which showed a statistically significant difference between the preoperative and postoperative data (*P* < 0.001). At the final follow up, the mean kyphosis of deformed complex vertebrae was 18.4° ± 2.29° (range, 13.8°—21.2°) without apparent loss of correction as compared with the postoperative data. The postoperative mean TK, LL, and SVA were reduced to 24.05° ± 3.84° (range, 17.1°-29.8°), 46.9° ± 3.53° (range, 41.3°-54.1°), and 0.6 cm ± 0.34 cm (range, 0.1 cm-1.2 cm), respectively. All the indexes also showed significant improvement compared with the preoperative data (*P* < 0.001 for all); there was no statistically significant difference between postoperative and final follow-up data (*P* > 0.05 for all). (Fig. 2) (Table 1).

**Table 1:** Preoperative, postoperative, and final follow-up measurement data

Case	Sex	Age (yrs)	Location of deformed vertebrae	Level of fusion	Period of follow up (months)	Duration of surgery (min)	Blood loss (ml)	Pre-OP							Post-OP				Final follow-up						
								Kyphosis of deformed vertebrae (°)	TK (°)	LL (°)	SVA (cm)	Frankel grade	VAS	ODI	Kyphosis of deformed vertebrae (°)†	TK (°)†	LL (°)†	SVA (cm)†	Kphosis of deformed vertebrae (°)‡ *	TK (°)‡ *	LL (°)‡ *	SVA (cm)‡ *	Frankel grade	VAS‡	ODI‡
1	F	5	T9-11	T7-12	30	185	450	81.2	91.3	85.7	4.3	D	3	28.9	20.3	27.5	47.9	0.9	18.4	29.8	55.3	1.4	E	0	2.2
2	F	8	T8-10	T7-12	24	310	690	89.8	85.7	87.4	4.9	E	4	24.4	21.2	26.4	50.2	0.8	20.6	26.1	49.7	0.7	E	1	6.7
3	F	10	T10-12	T8-L1	30	270	870	96.4	82.6	89.2	5.6	D	5	31.1	23.6	25.3	49.5	1.1	21.2	24.5	48.2	0.9	E	2	8.9
4	M	6	T7-8	T6-10	42	250	430	84.7	87.5	82.6	3.8	E	4	22.2	21.5	27.2	47.3	0.6	19.8	26.7	46.5	0.5	E	1	4.4
5	M	9	T11-12	T10-L2	30	230	660	77.2	71.6	69.1	2.9	E	3	17.7	18.4	21.7	44.8	0.4	17.5	22.4	45.1	0.5	E	1	4.4
6	F	5	T10-11	T9-12	48	240	410	79.4	73.2	77.4	3.2	E	3	20	19.7	22.6	45.4	0.6	17.6	23.2	45.7	0.4	E	1	4.4
7	F	11	T9-11	T8-L1	24	280	1010	102.6	97.4	93.8	6.8	D	6	33.3	22.5	29.8	54.1	1.2	20.3	27.6	51.8	0.8	E	2	11.1
8	M	7	T8-9	T7-11	36	250	530	85.9	89.7	84.3	4.1	E	4	24.4	22.1	27.7	48.6	0.7	20.7	26.9	48.2	1.2	E	1	6.7
9	F	10	T10-11	T9-L1	30	260	760	82.4	78.5	81.7	3.5	E	4	22.2	20.9	23.4	46.3	0.5	19.4	24.7	47.4	0.6	E	1	4.4
10	M	8	T10-12	T8-L1	30	250	740	90.5	83.2	86.9	5.1	D	5	26.7	21.8	24.6	46.7	0.8	19.9	23.4	46.3	0.9	E	1	6.7
11	M	6	T9-10	T8-12	36	230	470	75.7	79.9	74.3	2.7	E	3	15.6	17.6	20.8	43.9	0.2	16.2	21.3	44.5	0.3	E	1	4.4
12	F	11	T10-11	T8-12	24	210	890	74.1	71.3	75.8	1.9	E	2	13.3	16.9	19.5	43.2	0.3	15.6	20.1	43.6	0.2	E	0	2.2
13	M	7	T9-11	T7-12	36	260	640	87.9	81.5	79.6	4.6	D	4	28.9	21.4	28.2	51.6	0.7	19.7	27.3	50.9	0.6	E	1	4.4
14	M	6	T11-12	T10-L1	30	170	490	70.8	66.1	68.5	2.4	E	2	11.1	15.7	17.1	41.3	0.1	13.8	17.6	41.8	0.1	E	0	2.2
15	M	8	T9-10	T8-12	24	190	580	72.3	76.8	71.4	1.7	E	2	13.3	16.1	18.9	42.7	0.1	15.3	19.2	42.9	0.2	E	0	2.2
Mean	-	7.8	-	-	31.6	239	641.3	83.39	81.09	80.51	3.83	-	3.6	22.21	19.98	24.05	46.9	0.6	18.4	24.05	47.19	0.62	-	0.87	5.02
P (post-op / final follow-up vs. pre-op)								-	-	-	-	-	-	-	< 0.001	< 0.001	< 0.001	< 0.001	< 0.001	< 0.001	< 0.001	< 0.001	-	< 0.001	< 0.001
P (final follow-up vs. post-op)								-	-	-	-	-	-	-	-	-	-	-	< 0.001	0.982	0.604	0.754	-	-	-

*TK* Thoracic kyphosis, *LL* Lumbar lordosis, *SVA* Sagittal vertical axis, *VAS* Visual analogue scale, *ODI* Oswestry disability index

The postoperative and preoperative data as well as the final follow-up and preoperative data were analyzed using paired t tests

*P* < 0.05 implies statistically significant difference

^†^*P* < 0.05 (postoperative vs. preoperative)

^‡^*P* < 0.05 (final follow-up vs. preoperative)

^*^P (final follow-up vs. postoperative)

### VAS and ODI

VAS and ODI were assessed preoperatively and at the final follow up. At the final follow up, VAS score reduced from 3.6 ± 1.18 (range, 2–6) to 0.87 ± 0.64 (range, 0–2); and ODI score reduced from 22.21 ± 6.93 (range, 11.1–33.3) to 5.02 ± 2.6 (range, 2.2–11.1); they both revealed significant statistical differences between final follow-up and preoperative scores (*P* < 0.001).

## Discussion

In this study, severe thoracic post-tubercular angular kyphosis was involved with two or more fused vertebrae, and the preoperative mean kyphosis of deformed complex vertebrae was 83.39°. Given these characteristics of this group of patients account, it was difficult to choose an appropriate resection area and find a proper osteotomy technique that could be safe and effective. Therefore, DCVO was performed at the apex of kyphotic deformity. A wider posterior wedge-shaped, three-column and grade 4/5 spinal osteotomy was performed within deformed complex vertebrae. The upper and lower endplates of the deformed complex vertebrae were preserved to prevent growth imbalance of the anterior and posterior columns in children during their growth phase by keeping the growth potentials of the anterior column and to reduce surgical trauma and intraoperative blood loss. The anterior longitudinal ligament served as a hinge for the bone-to-bone closure of the gap. In this group of cases, the nerve roots were not ligated and slightly affected the visibility of the operative field and the freedom of osteotomy. However, there was no direct damage to the nerve roots, and this helped preserve nervous system functional integrity.

After DCVO, the postoperative mean kyphosis was reduced to 19.98° with a kyphosis correction of 63.41°. The correction rate was considerably better than that of PSO and VCR reported by some authors [9, 10, 12, 14, 17, 18]. Patients' body figure, sagittal alignment and balance (TK, LL, and SVA), pain (VAS), daily activities (ODI), and neurological function (Frankel's grade) all showed good improvement. However, the operation time, blood loss, difficulty of operation, incidence of complications, and neurological events were significantly lower than those of VCR reported in previous studies [12, 14, 17, 19, 20].

Although conservative treatment is effective with the use of antituberculosis medication, however, it is well known that progressive collapse of anterior spinal elements may still occur under gravity and compression. Moreover, due to eccentric loads and spinal growth imbalance of anterior and posterior columns in children, the wedge shape of the vertebral body and kyphotic deformity could be aggravated. Neurological deterioration, cardiopulmonary dysfunction, and severe persistent back pain are all possible side effects of post-tubercular kyphosis [2, 21, 22]. As a result, children with severe thoracic post-tubercular angular kyphosis frequently require surgery.

The primary goal of surgery is to treat spinal deformity and prevent further deformity, as well as to restore and reconstruct the physiological sagittal alignment and decompress the nerve to improve its function. To date, numerous techniques have been developed to treat kyphosis secondary to spinal tuberculosis, mainly including Smith-Petersen Osteotomy (SPO), PSO, and VCR [10, 14, 21, 23]. However, none of those mentioned above techniques was considered a golden criterion for attaining minor trauma and significant correction.

SPO could only achieve approximately 10.7° per segment of kyphosis correction. However, after opening the anterior column, it was determined that there was a risk of permanent neurological impairment and aortic rupture [10, 24]. In recent years, three-column osteotomy has been the primary technique for the treatment of severe angular kyphosis. Hu et al. [9] compared SPO and PSO in the treatment of rigid thoracolumbar kyphotic deformity, finding that PSO's kyphosis correction ranged from 31.7° to 48° with an average of 36.7°, while SPO's was 8.74° less. Furthermore, the incidence of biomechanical complications, including instrument breakage, anterior cortex fracture, pedicle screw loosening, pedicle fraction, vertebral body translation, and nonunion, in PSO was lower than in SPO [10, 25]. In addition, a retrospective study of patients with rigid post-tuberculous kyphosis and no neurological deficit preoperatively, whose mean kyphotic angle was 58.8°, revealed the mean kyphosis correction of only 44.2° after PSO [18].

However, there are several disadvantages of PSO for the treatment of severe thoracic angular kyphosis (> 70°) [13, 18, 26]: (1) The PSO technique only achieves limited deformity correction and cannot achieve the goal of treating severe angular kyphosis of more than 70°. (2) Severe post-tubercular angular kyphosis commonly involves two or more deformed fused vertebrae. Thus, defined PSO in one vertebra may not accord with the requirements of appropriate resection area wedge osteotomy at the apex of kyphosis. However, multiple deformed and fused vertebrae were treated as a single unit, according to DCVO, and a wider posterior wedge-shaped and three-column osteotomy was performed within multiple deformed and fused vertebrae to correct a more extensive range of angles.

The most effective therapeutic technique is VCR, and 3-column circumferential vertebral osteotomy created a segmental defect with apparent instability, necessitating temporary instrumentation. Following that, the two divided parts were brought together for rectification and realignment, which necessitated titanium mesh or block bone grafts. Suk et al. [17] reported seventy patients with severe spinal deformity were treated by posterior VCR, and the deformity correction was 45.2° in the sagittal plane and 61.9% in the coronal plane. The mean blood loss was 2980 ml in the postinfectious kyphosis group, and 24 patients suffered severe complications.

However, in a study of 147 patients with severe spinal deformity who underwent VCR, Lenke et al. [14] discovered that these complex reconstructions were associated with a 59% complication rate, with 39 cases (27%) suffering an intraoperative neurological event, such as a failed wake-up test or a change in spinal cord monitoring. Hua et al. [20] treated severe post-tubercular kyphosis with VCR: 2 of 13 cases had spinal cord injuries, including one complete paraplegia and one incomplete paraplegia, and a total neurological complication rate of about 15.4%; the mean operative time and blood loss were 388 min and 2554 ml. Other complications, including fixation failure due to nonunion or pseudoarthrosis, hematomas, hemopneumothorax, and infections, also occurred frequently [12, 14, 17, 19]. Therefore, VCR was an exhausting and technically demanding procedure with possible risks of significant complications. This point of view was commonly recognized as a consensus by most surgeons.

Therefore, when compared to PSO and VCR, the following characteristics and benefits of DCVO can be summarized [8–20, 26]: (1) Deformed complex vertebrae usually involve two or more vertebral bodies and the partial or total fusion of many segments' facet joints and intervertebral discs. Multiple deformed and fused vertebrae were treated as a single unit, which simplified complicated problems and aided in developing a more specific osteotomy within the unit and precise positioning during surgery. (2) A wider posterior wedge-shaped and three-column osteotomy was performed within deformed complex vertebrae to correct a larger range of angles. The upper and lower endplates and the part of cancellous bone that adjoined normal vertebrae were preserved without involving normal discs. Thus, beneficial for reducing surgical trauma and intraoperative blood loss. (3) Anterior longitudinal ligament was also kept as a hinge for closure of the gap. The spine structure was relatively stable, and closure resistance was lower, which reduces the risk of displacement. (4) Bone-to-bone closure was achieved in the anterior and middle columns without interbody fusion cages, resulting in better fusion efficiency. (5) After DCVO, this group of patients had a kyphosis correction of 63.41°, which was more significant than the PSO correction rate. (6) It was effective to treat kyphosis and prevent growth imbalance of the anterior and posterior columns in children during their growth phase by keeping the growth potentials of the anterior column without destroying the upper and lower endplates. (7) DCVO combined the benefits of both PSO (hinge for bone-to-bone closure) and VCR (maximal correction of a wide range of angles). While also avoiding the drawbacks of both PSO (in one vertebra with restricted and lower correction rate) and VCR (greater incidence of complications, blood loss, and difficulty of operation; longer operation time and neurological sequelae).

Some limitations need to be taken into account. The included cases had a small sample size, and the long-term follow-up outcome should be investigated further. Future prospective comparative studies may provide further insight into the advantages and potential fallacies of these procedures.

## Conclusions

In conclusion, DCVO was an individualized osteotomy for treating severe thoracic post-tubercular angular kyphosis in children. It could be safe and effective in reducing the incidence of complications and significantly improving kyphosis correction.

## Data Availability

The datasets analyzed during the current study are not publicly available because a further study about severe angular kyphosis is in progress in our institution, but are available from the corresponding author on reasonable request.

## Abbreviations

DCVO: Deformed complex vertebral osteotomy
SVA: Sagittal vertical axis
VAS: Visual Analogue Scale
ODI: Oswestry Disability Index
CT: Computed tomography
MRI: Magnetic resonance imaging
TK: Thoracic kyphosis
LL: Lumbar lordosis
MEP: Motor evoked potential
SEP: Somatosensory evoked potential
PCO: Posterior column osteotomy
SPO: Smith-Petersen Osteotomy
PSO: Pedicle subtraction osteotomy
VCR: Vertebral column resection
CRP: C-reactive protein
ESR: Erythrocyte sedimentation rate
